# *Acetobacter cibinongensis* Bacteremia in Human

**DOI:** 10.3201/eid1305.060532

**Published:** 2007-05

**Authors:** Anne Gouby, Corinne Teyssier, Frédérique Vecina, Hélène Marchandin, Céline Granolleras, Isabelle Zorgniotti, Estelle Jumas-Bilak

**Affiliations:** * Hôpital Caremeau, Nîmes, France; † Université Montpellier 1, Montpellier, France; ‡ Hôpital Arnaud de Villeneuve, Montpellier, France

**Keywords:** *Acetobacter*, *Acetobacter cibinongensis*, acetic acid bacteria, Alpha Proteobacteria, bacteremia, hemodialysis, 16S rRNA, phylogeny, letter

**To the Editor:** The genus *Acetobacter* belongs to the group of acetic acid bacteria that oxidize alcohols or sugars incompletely, leading to the accumulation of acetic acid. Acetic acid bacteria are of great industrial interest because of their use to produce vinegar from spirits, wine, beer, and cider in temperate regions of Europe, the Americas, and Japan. Several species seem to be associated with tropical climates. In Southeast Asia, *Acetobacter* spp. have been found in fermented foods such as tea fungus beverage, palm vinegar, palm wine, nata de coco, and pickles (*1*). *A. cibinongensis* is mainly found in tropical fruits and flowers (*2*). We describe a case of human infection with a member of the genus *Acetobacter*.

The patient was an HIV-seronegative, 40-year-old man who for 1 year had been receiving chronic hemodialysis for end-stage renal failure. He had a history of intravenous drug use, and continued use was suspected. In February 2005, when admitted for a routine dialysis session, he had fever (38°C) and bronchitis and was receiving empiric treatment with amoxicillin (2 g/day). His respiratory status improved slightly, but fever persisted after 48 hours. On his right forearm, he had a large inflammatory skin lesion that followed the course of an arteriovenous fistula, suggestive of staphylococcal infection. Treatment was switched to pristinamycin (2 g/day for 4 days). The patient’s leukocyte count was within normal limits, but his C-reactive protein level was elevated (50 mg/L). Two blood samples were drawn, 1 through a subclavian catheter implanted in 2004 and the other through the arteriovenous fistula. After 4 days, a gram-variable polymorphic rod, named nîmes373, grew from both aerobic culture vials and yielded very small polymorphic colonies on Columbia sheep blood and blood-chocolate agar plates (Bio-Rad, Marnes-la-Coquette, France). Enhanced growth was observed on *Legionella* agar (AES Chemunex, Bruz, France) and on R2A agar plates (BD-BBL, Le Pont de Claix, France). Biotyping of this catalase-positive, oxidase-negative rod with API 20NE and API 20E strips (bioMérieux, Marcy l’Etiole, France) and with the ID-GNB card system on a VITEK 2 apparatus (bioMérieux) did not identify the bacterium. Positive reactions were obtained for acetoin production and citrate assimilation. Antimicrobial drug–susceptibility pattern could not be validated owing to the lack of interassay reproducibility. Pristinamycin was replaced by broad-spectrum antimicrobial therapy consisting of cefazolin (1 g every 48 hours) and tobramycin (225 mg after each hemodialysis session). After 3 courses of this regimen, treatment was changed to amoxicillin (2 g/day, plus 1 g after each dialysis session) for 4 weeks. The local inflammation and fever subsided, the C-reactive protein level returned to normal, and the patient’s clinical status improved with no recurrence of infection.

A 1389-bp 16S rDNA sequence was obtained for strain nîmes373, as described (*3*). The sequence matched those of *A. cibinongensis* deposited in the GenBank database (>99.6% identity). The 16S rDNA-based phylogeny confirmed the affiliation of strain nîmes373 to the species *A. cibinongensis* in the family *Acetobacteraceae* (Figure). The growth of nîmes373 on 0.7% calcium carbonate agar plates with 7% ethanol (pH 3.5) cleared calcium carbonate. This indicated tolerance of the isolate to acidic conditions and ability to produce organic acids from alcohol, thereby proving its affiliation to the acetic acid bacteria group.

**Figure:**
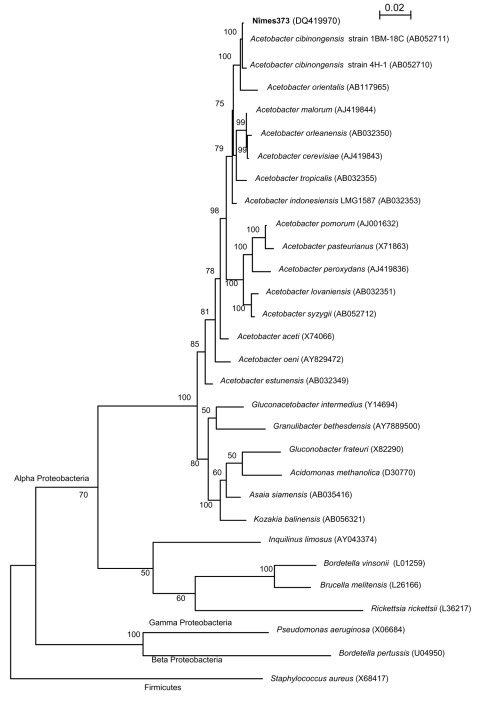
16S rDNA maximum-likelihood phylogenetic tree showing the relationships of the isolate nîmes373 with 15 species of the genus *Acetobacter* and 6 strains representative of the 6 other genera of acetic acid bacteria in the Alpha Proteobacteria. Sequences of Alpha, Beta, and Gamma Proteobacteria of clinical relevance were also included in the tree*. Staphylococcus aureus* (*Firmicutes*) 16S rDNA was used as an outgroup. The 16S rDNA sequences used to reconstruct this tree were obtained from the GenBank database, and their accession numbers are indicated in brackets. The tree was reconstructed using DNAML from the PHYLIP package v. 3.6.6, on the basis of the F84 (+ gamma distribution + invariant sites) substitution model. The scale bar indicates 0.02 substitutions per nucleotide position. Numbers given at the nodes represent bootstrap percentages calculated on 100 replicates.

Currently, 6 genera of acetic acid bacteria are recognized in the Alpha Proteobacteria lineage (Figure). Only the main genera (*Acetobacter*, *Gluconacetobacter*, *Gluconobacter*) are discriminated by analytic methods, such as high-performance liquid chromatography, which are not used in routine microbiology (*4*). The tree showed that 16S rDNA sequencing was effective for genus and species identification of acetic acid bacteria (Figure). This method is also rapid and convenient for use in a medical microbiology laboratory.

Bacteria have been used in food processing and have thus been ingested, live or dead, throughout human history. The safety of lactic and acetic acid bacteria has been confirmed over the years (*5*). However, in recent years, lactic acid bacteria belonging to the genera *Lactobacillus*, *Leuconostoc*, *Pediococcus*, or *Bifidobacterium* have been isolated from a variety of clinical samples, generally from patients with diabetes, cancer, or immunosuppression (*6*). As lactic acid bacteria are part of the normal human flora, their clinical relevance is often difficult to establish. In contrast, acetic acid bacteria have never been isolated from human flora; recently, the pathogenicity of an unknown bacterium that probably belonged to acetic acid bacteria, *Granulobacter* *bethesdensis,* was found in a patient with lymphadenitis (*7*).

Our patient denied travel outside France and had no exotic dietary habits. He was probably infected by direct inoculation, possible during intravenous drug injection or after unnoticed injury when he worked in vineyards. For hemodialysis patients, vascular access infections are a major cause of illness and death (*8*). Also for these patients, unusual bacteria with low inherent pathogenicity are increasingly reported as potential causes of bacteremia (*9*). The decreased phagocytic activity of neutrophils and monocytes associated with chronic renal failure may increase susceptibility to bacteria that would otherwise have low pathogenicity (*10*). This second known report of human infection with acetic acid bacteria should alert clinicians to the risk for opportunistic infections with these bacteria, which are broadly used in food processing.
